# Molten‐Salt‐Assisted Chemical Vapor Deposition Process for Substitutional Doping of Monolayer MoS_2_ and Effectively Altering the Electronic Structure and Phononic Properties

**DOI:** 10.1002/advs.202001080

**Published:** 2020-07-01

**Authors:** Wei Li, Jianqi Huang, Bo Han, Chunyu Xie, Xiaoxiao Huang, Kesong Tian, Yi Zeng, Zijing Zhao, Peng Gao, Yanfeng Zhang, Teng Yang, Zhidong Zhang, Shengnan Sun, Yanglong Hou

**Affiliations:** ^1^ Department of Materials Science and Engineering, College of Engineering Peking University Beijing 100871 China; ^2^ Beijing Key Laboratory for Magnetoelectric Materials and Devices (BKL‐MMD) Beijing Innovation Center for Engineering Science and Advanced Technology (BIC‐ESAT) Beijing 100871 China; ^3^ Electron Microscopy Laboratory and International Center for Quantum Materials, School of Physics Peking University Beijing 100871 China; ^4^ Shenyang National Laboratory for Materials Science, Institute of Metal Research Chinese Academy of Sciences Shenyang 110016 China; ^5^ Collaborative Innovation Center of Quantum Matter Beijing 100871 China

**Keywords:** 2D, molten‐salt‐assisted chemical vapor deposition, molybdenum disulfide, substitutional doping

## Abstract

Substitutional doping of layered transition metal dichalcogenides (TMDs) has been proved to be an effective route to alter their intrinsic properties and achieve tunable bandgap, electrical conductivity and magnetism, thus greatly broadening their applications. However, achieving valid substitutional doping of TMDs remains a great challenge to date. Herein, a distinctive molten‐salt‐assisted chemical vapor deposition (MACVD) method is developed to match the volatilization of the dopants perfectly with the growth process of monolayer MoS_2_, realizing the substitutional doping of transition metal Fe, Co, and Mn. This doping strategy effectively alters the electronic structure and phononic properties of the pristine MoS_2_. In addition, a temperature‐dependent Raman spectrum is employed to explore the effect of dopants on the lattice dynamics and first‐order temperature coefficient of monolayer MoS_2_, and this doping effect is illustrated in depth combined with the theoretical calculation. This work provides an intriguing and powerful doping strategy for TMDs through employing molten salt in the CVD system, paving the way for exploring new properties of 2D TMDs and extending their applications into spintronics, catalytic chemistry and photoelectric devices.

## Graphical abstract

An intriguing and powerful molten‐salt‐assisted chemical vapor deposition strategy is developed to achieve effective metal substitutional doping of monolayer MoS_2_. In this process, the salt with low melting point promotes the volatilization of the dopant precursors during the growth of monolayer MoS_2_, which creates excellent conditions and opportunities for heteroatoms (Fe, Co, and Mn) incorporation.

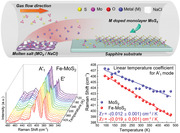

## 1 Introduction

2D transition metal dichalcogenides (TMDs), as a new class of layered materials with atomic thickness, have provided a fertile ground for the research on fundamental physical phenomena and future promising applications.^[^ ^1^, ^2^, ^3^, ^4^, ^5^, ^6^ ^]^ In terms of their intrinsic chemical compositions and structures, these materials exhibit widely diverse properties, varying from semiconducting (e.g., MoSe_2_)^[^ ^7^ ^]^ to superconducting (e.g., NbSe_2_),^[^ ^8^ ^]^ nonmagnetic (e.g., MoS_2_)^[^ ^9^ ^]^ to ferromagnetic (e.g., VSe_2_),^[^ ^10^ ^]^ wide‐bandgap (e.g., WS_2_)^[^ ^11^ ^]^ to narrow‐bandgap (e.g., PtSe_2_).^[^ ^12^ ^]^ Since the characteristics presented by a specific material are extremely monotonous and limited, controllably tuning its intrinsic properties or even endowing it with some new fascinating features distinguishing from its original ones is of great significance for extending their applications.

To address the need mentioned above, enormous efforts have been taken in engineering the structures of pristine TMDs. Initial attempts focused on removing some specific atoms from the skeleton of TMDs for creating vacancy defects.^[^ ^13^, ^14^, ^15^ ^]^ Nevertheless, the properties induced by vacancies will be vanished easily by local structural rearrangements during annealing operation or chemical passivation process.^[^ ^16^ ^]^ Aside from creating vacancy defects, heteroatom doping is an another effective strategy to regulate and control the intrinsic properties of TMDs, and has aroused tremendous research interests recently.^[^ ^17^, ^18^, ^19^, ^20^ ^]^ More intriguingly, due to their abundant d‐orbital electrons, the substitutional doping of the pristine TMDs by transition metal atoms can bring about a lot of properties, which are not presented in them intrinsically, such as magnetism,^[^ ^21^, ^22^ ^]^ photoresponse,^[^ ^23^, ^24^ ^]^ and conductivity.^[^ ^25^ ^]^ For example, Re‐doped MoSe_2_ nanoflakes have been prepared by CVD, this monolayer 2D alloys exhibited ferromagnetism even at room temperature based on the zero‐field cooled and field cooled measurements.^[^ ^26^ ^]^ Besides, monolayer W*_x_*Mo_1−_ *_x_*S_2_ was designed and synthesized by sulfurizing the MoO_3_ and WO_3_ at the same time. The alloyed W*_x_*Mo_1−_ *_x_*S_2_ displayed various positions of PL emission peaks depending on the amount of W composition.^[^ ^27^ ^]^ Unfortunately, in the past few years, most reported achieved transition‐metal substitutions in monolayer TMDs were limited to Mo,^[^ ^28^ ^]^ W,^[^ ^29^ ^]^ Re,^[^ ^30^ ^]^ and Nb,^[^ ^31^ ^]^ which can form alloy phase with each other since their similar structures and properties. When it comes to other transition metals, only a few elements have been doped in monolayer TMDs successfully.^[^ ^32^ ^]^ Therefore, at present, a simple but effective strategy for metal substitutional doping of monolayer TMDs is extremely desired for the fundamental research and future applications.

In this work, we propose a molten‐salt‐assisted chemical vapor deposition (MACVD) method to tactfully match the volatilization of the dopant sources with the growth of TMDs, achieving the controllable synthesis of Fe‐doped monolayer MoS_2_ nanoflakes in one step, this method can be extended to Co and Mn as well. In addition, X‐ray photoelectron spectroscopy (XPS) and high‐angle annular darkfield scanning transmission electron microscopy (HADDF‐STEM) are utilized to certify the existence of the heteroatoms and demonstrate that the transition metal atoms substitute the position of the Mo atoms. It is worth mentioning that this doping strategy effectively alters the electronic structure and phononic properties of the pristine MoS_2_. Moreover, temperature‐dependent Raman spectrum is employed for the first time to explore the effect of the Fe dopants on the first‐order temperature coefficient of monolayer MoS_2_. Furthermore, this doping effect has been understood quantitatively from the theoretical perspective.

## 2 Results and Discussion

**Figure 1:**
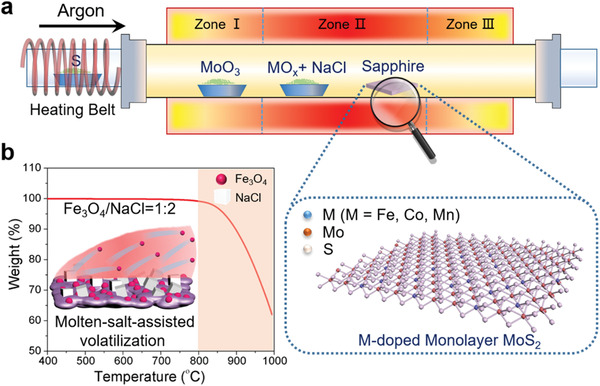
a) Schematic illustration of the CVD setup for the synthesis of transition metal‐doped monolayer MoS_2_ nanoflakes on sapphire substrate with the addition of metal oxide (MO*_x_*) and NaCl as the molten salt. b) The thermogravimetric curve of the mixed Fe_3_O_4_ and NaCl at the mass ratio of 1:2, which indicates Fe precursors start to volatilize dramatically above 800 °C assisted by molten‐salt.

The transition metal‐doped monolayer MoS_2_ nanoflakes were achieved by the MACVD process. **Figure** **1**a illustrates the designed three‐zone CVD furnace set up, in which MoO_3_ was vaporized sustainably at 530 °C in the first zone of the furnace. Aiming to dope heteroatoms into the lattice of MoS_2_ effectively, we precisely controlled the volatilization temperature of the metal precursors within the growth process of monolayer MoS_2_. Specifically, considering that some salts (e.g., NaCl and KI) can dramatically decrease the melting point of metal oxide precursors,^[^ ^33^, ^34^ ^]^ we introduced NaCl to the precursors, mixed with metal oxide (MO*_x_*) as the mass ratio of 2:1 and placed 20 mg of this mixture just upstream of the sapphire substrate in the second zone of the furnace. By taking Fe‐doped MoS_2_ (Fe‐MoS_2_) as an example, according to the thermogravimetric curve in Figure 1b, we can see that the Fe precursors start to volatilize dramatically above 800 °C, that is, Fe dopants will be brought to the surface of the sapphire substrate by the 50 sccm argon (Ar) gas during the growth of the monolayer MoS_2_ at 870 °C, which creates excellent conditions and opportunities for Fe incorporation into the lattice of MoS_2_. In this synthesis process, the chamber was kept in low pressure (≈30 Pa), and when the temperature of the first zone reached 400 °C, S vapors were introduced into the furnace with heating belts.

**Figure 2:**
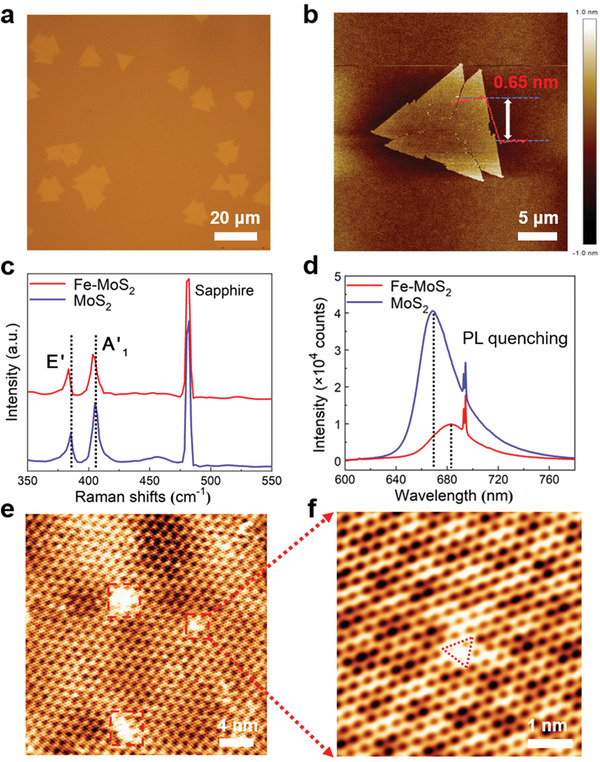
a) Optical image of the obtained Fe‐MoS_2_ nanoflakes. b) AFM image and height profile of Fe‐MoS_2_ nanoflakes. c,d) Raman and PL spectra of Fe‐MoS_2_ and pure MoS_2_, respectively. e,f) Scanning tunneling micrograph of Fe‐MoS_2_ nanoflakes on HOPG.

After growth, 2D nanoflakes with sawtooth corners can be found in the sapphire substrate, as shown in **Figure** **2**a, the lateral size of which is around 10 µm. In contrast, if Fe precursors were not included in the furnace while other experiment parameters remained unchanged, regular triangular MoS_2_ nanoflakes were obtained (Figure S1a, Supporting Information). This phenomenon can be attributed to the doping of heteroatoms, which can change the growth kinetics during the synthesis of monolayer MoS_2_.^[^ ^35^ ^]^ Atomic force microscope (AFM) measurements reveal that the thicknesses of Fe‐MoS_2_ and pure MoS_2_ nanoflakes are around 0.65 nm (Figure 2b) and 0.52 nm (Figure S1b, Supporting Information), corresponding to the feature of the monolayer.^[^ ^36^ ^]^ Figure 2c shows that the two characteristic Raman peaks of pure MoS_2_ at room temperature, A'_1_ (out‐of‐plane vibrational mode) and E' (in‐plane vibrational mode) are displayed at 405.2 and 385.4 cm^−1^. In Fe‐MoS_2_ nanoflakes, the A'_1_ and E' peaks shift to 404.1 and 383.8 cm^−1^, respectively. This redshift of characteristic Raman peaks by doping transition‐metal atoms was reported before, but the intrinsic mechanism is not clearly explained,^[^ ^37^, ^38^ ^]^ which will be discussed later in detail. In addition, distinct broadening of peaks and weakening in peak intensities are also related to the doping effect.^[^ ^39^ ^]^ Meanwhile, we can see that the frequency differences of around 20 cm^−1^ between the two dominant peaks before and after doping are in agreement with that of the monolayer, providing an additional evidence of the monolayer feature of the synthesized nanoflakes.^[^ ^36^ ^]^ Furthermore, the PL spectrum at room temperature (Figure 2d) is quenched notably in intensity and the emission wavelength is red‐shifted after doping, which indicates that the addition of Fe dopants in MoS_2_ leads to the increase of trions concentration by electron doping, thus enhancing the nonradiative recombination and shortening the bandgap of pristine MoS_2_.^[^ ^35^ ^]^ Taking a step further, atomic‐resolution scanning tunneling micrograph (STM) was employed to unveil the existence of the heteroatoms. As shown in Figure 2e, we can see that there are some bright regions scattered on the STM image of Fe‐MoS_2_, while the Moiré pattern of the pure MoS_2_ (Figure S2, Supporting Information) is extremely uniform, which is similar to the case of Re doped monolayer MoS_2_.^[^ ^40^ ^]^ Since the STM experiment was performed on Fe‐MoS_2_ on top of Highly Oriented Pyrolytic Graphite (HOPG), we set up Fe‐MoS_2_ on top of graphene for simulation as shown in Figure S3a (Supporting Information). The simulated STM image in Figure S3b (Supporting Information) indicates that the bright spots are from S atoms in the vicinity of Fe atom. The brightness (or the height of the iso‐surface) decreases for S atoms which go away from the Fe atom. The triangle shape of the bright spot is consistent with the experimental observation (Figure 2f).

**Figure 3:**
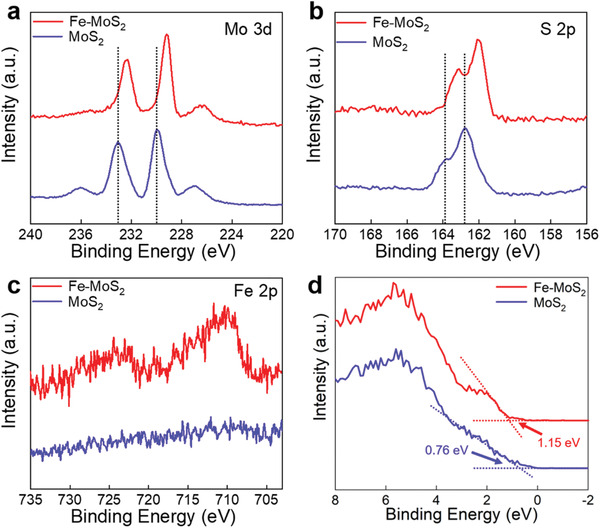
a–c) X‐ray photoelectron spectroscopy (XPS) scans of Mo 3d (a), S 2p (b), and Fe 2p (c) core‐level binding energies of Fe‐MoS_2_ and pure MoS_2_, calibrated by the adventitious carbon C 1s peak. d) Valence band maxima (VBM) comparison between Fe‐MoS_2_ and pristine MoS_2_.

The Fe dopants in the MoS_2_ nanoflakes are further analyzed with XPS. **Figure** **3**a,b displays the comparisons of XPS results measured from the Fe‐MoS_2_ (red solid line) and pure MoS_2_ (blue solid line). Compared with the XPS peaks of Mo and S of the pristine MoS_2_, those in the Fe‐MoS_2_ nanoflakes present uniform shifts around 0.7 eV toward lower binding energy, revealing the changes in chemical microenvironment of Mo and S atoms, and the Mo—S bond strength has been weakened after Fe doping. As shown in Figure 3c, the obvious binding energy peaks related to the Fe 2p at 710.3 and 724.4 eV are discovered only in the Fe doped MoS_2_ samples.^[^ ^41^ ^]^ Moreover, the Fe dopants in the MoS_2_ also shift the valence band edge (Figure 3d) by 390 meV from 0.76 to 1.15 eV, revealing that the electronic structure of the monolayer MoS_2_ has been altered by the addition of Fe.

**Figure 4:**
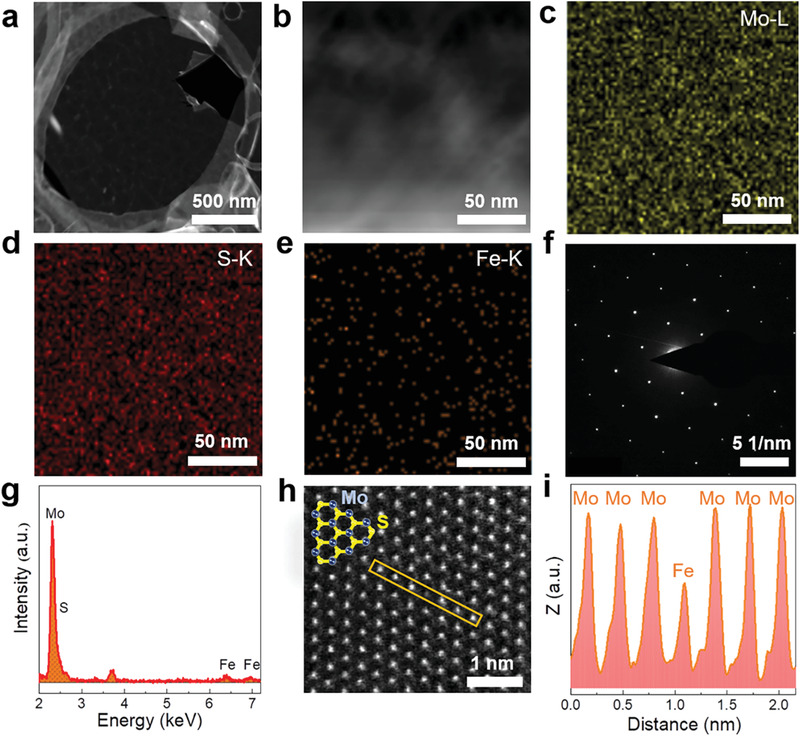
a,b) HAADF‐STEM images of Fe‐MoS_2_ nanoflakes transferred onto Cu grids. c–e) EDX elemental mapping images of the sample shown in (b). f) Corresponding selected area electron diffraction pattern of Fe‐MoS_2_ nanoflakes. g) EDX spectrum of the Fe‐MoS_2_ nanoflakes. h) High‐resolution HADDF‐STEM image and i) the intensity spectra of the selected area of monolayer Fe‐MoS_2_ in (h).

To ulteriorly confirm the presence and substitutional doping of the Fe atoms, we transferred the Fe‐MoS_2_ nanoflakes from the sapphire substrates onto Cu grids for transmission electron microscopy (TEM) characterization. **Figure** **4**a shows that the HAADF‐STEM image of Fe‐MoS_2_, revealing that the layered feature is retained when some Fe atoms are incorporated into monolayer MoS_2_. Moreover, the energy‐dispersive X‐ray (EDX) spectroscopy in Figure 4g reveals that the chemical composition of the sample includes not only Mo and S but also Fe elements. The corresponding EDX element mapping (Figure 4b,e) shows that Fe atoms are distributed homogeneously on the plane of MoS_2_ nanoflakes. Meanwhile, the weaker brightness and lower density compared with the mapping images of Mo and S indicate that the amount of doped Fe atoms is limited. Furthermore, the selected area electron diffraction (SAED) pattern in Figure 4f also confirms the hexagonal single‐crystal structure is preserved after doping. To identify the location of Fe atoms within the monolayer MoS_2_, high‐resolution HADDF‐STEM measurement was performed. Since there is a high positive correlation between HAADF intensity and atomic number, the Fe atoms (*Z* = 26) will display lower HAADF intensity compared with Mo (*Z* = 42). From Figure 4h and Figure S4 (Supporting Information), we can find that some darker spots randomly distributed on the plane of MoS_2_. The corresponding HAADF intensity of the atoms contrasted in **Figure** 4i reveals that the Fe atoms substitute for Mo atoms instead of being adsorbed on the surface of MoS_2_. Furthermore, the concentration of the Fe dopants within the monolayer MoS_2_ is estimated to be around 4.49 at% according to the statistic results of the high‐resolution HADDF‐STEM image (Figure S4, Supporting Information).

**Figure 5:**
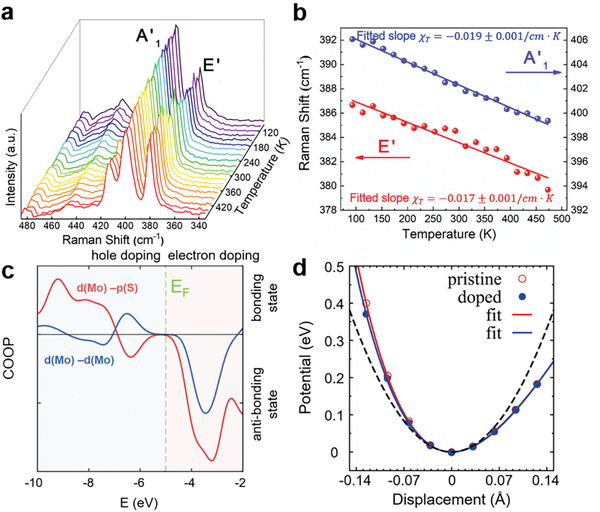
a) Raman spectra of monolayer Fe‐MoS_2_ collected at various temperatures between 93 to 473 K. b) Raman peak frequencies of both A'_1_ (blue circles) and E' (red circles) modes as a function of temperature. c) Crystal orbital overlapping population (COOP) of MoS_2_ monolayer. Red and blue solid lines represent the bonding character between two dominating Mo‐d orbital and S‐p orbital, respectively. Positive (negative) COOP shows the bonding (anti‐bonding) states. d) Vibrational potential versus atomic displacement curves of the A'_1_ mode. Dots are the calculation data, and solid lines are the fitting using the Morse potential. The dashed black line is for a perfect harmonic potential.

The temperature‐dependent Raman measurements were conducted for the first time to research the effect of Fe dopants on the first‐order temperature coefficient of monolayer MoS_2_. **Figure** **5**a shows a series of Raman spectra obtained from monolayer Fe‐MoS_2_ nanoflakes when the temperature of the test chamber changes from 93 to 473 K. With the temperature increasing, both characteristic peaks A'_1_ and E' display an expected red‐shift.^[^ ^42^ ^]^ In addition, the absolute frequency shift value of the A'_1_ mode is larger than that of the E' mode, implying that A'_1_ mode possesses a stronger phonon‐phonon scattering than the E' mode.^[^ ^43^ ^]^ Figure 5b presents the temperature dependence of the Raman peak position from 93 to 473 K for the A'_1_ (blue circles) and E' (red circles) modes. The variation of the peak frequency *ω* (cm^−1^) as a function of the absolute temperature (*T*) evolution followed a linear relationship Δ*ω* = *ω*(*T*_2_) − *ω*(*T*_1_) = *χ*_*T*_(*T*_2_ − *T*_1_) = *χ*_*T*_Δ*T*, in which *χ*_T_ is the first‐order temperature coefficient of the corresponding modes.^[^ ^44^ ^]^ By using the linear fitting, the linear temperature coefficients *χ*_T_ are − 0.017  ±  0.001 cm^−1^ K^−1^ and − 0.019  ±  0.001  cm^−1^ K^−1^ for the E' and A'_1_ vibrational modes, extracted from the slopes respectively. Similarly, we obtain the linear temperature coefficients *χ*_T_ are − 0.009  ±  0.001 and − 0.012  ±  0.001  cm^−1^ K^−1^ for the E' and A'_1_ vibrational modes of pure MoS_2_ according to the identical temperature‐dependent Raman measurement (Figure S5, Supporting Information). As a consequence, the linear temperature coefficients *χ*_T_ (both E' and A'_1_ modes) of Fe‐MoS_2_ nanoflakes become larger compared with that of the pure MoS_2_, which is ascribed to the electron‐doping induced enhancement of phonon–phonon scattering, as discussed later in the manuscript.

To understand the substitutional effect of Fe for Mo on Raman spectra of MoS_2_, we had the following theoretical analysis. First, we calculated the crystal orbital overlapping population (COOP) of pristine MoS_2_, as shown in Figure 5c. From the COOP, the main bonding states take place between Mo‐d and S‐p orbitals, and also between Mo‐d and neighboring Mo‐d orbitals. Positive and negative values of COOP indicate the bonding and anti‐bonding characters, respectively. Additional electrons when substituting Mo (d^5^s^1^) with Fe (d^6^s^2^) will occupy the Mo‐S anti‐bonding states, as seen from Figure 5c, which is expected to weaken the bonding strength (the Hooks coefficient *k*) between Mo and S and therefore induce a redshift of both A'_1_ and E' peaks $\omega \propto \sqrt{\frac{k}{\mu }}$, in which *μ* is the reduced mass of the Raman mode, *k* ↓, *ω* ↓). This explanation can find additional support from a gradual red shift of Raman peaks from Mn‐, Fe‐ and Co‐substituted MoS_2_ in Table S1 (Supporting Information), due to the increasing electronic doping effect from Mn (d^5^s^2^) via Fe (d^6^s^2^) to Co (d^7^s^2^). Then, treating Fe‐MoS_2_ as electron‐doped MoS_2_, we calculated the Raman spectra of both pristine and electron‐doped MoS_2_, as shown in Figure S6a (Supporting Information). From the calculated Raman spectra, both A'_1_ and E' peaks indeed show a red‐shift from the pristine to electron‐doped MoS_2_. Last, to comprehend the temperature dependence of frequency, we calculated the inter‐sulfur atomic potential as a function of atomic displacement for the A'_1_ mode in Figure 5d (E' is similar as shown in Figure S6b, Supporting Information). It is evident that the potentials compared with the perfect harmonic potential (black dashed line in Figure 5d) show some anharmonicity, and become more shallow from the pristine to electron‐doped MoS_2_. The Morse‐type function, for example, $V\;\left(x\right)=a{\left(1-e^{-b(x-x_{0})}\right)}^{2}\;$ can be used to fit the calculated potential very well. By Taylor‐expanding the Morse‐type potential and keeping the high‐order term up to the 4th order, we will see that the anharmonicity is intimately relevant to the fitting parameter *b*. A reduced *b* value from the pristine to electron‐doped MoS_2_ indicates an increased anharmonicity, which gives rise to a stronger temperature dependence of Raman frequency in the electron‐doped system than the pristine MoS_2_, as observed experimentally in Figure 5a,b, and Figure S5 (Supporting Information).

Following the proposed MACVD strategy, Co or Mn‐doped monolayer MoS_2_ nanoflakes can also be synthesized by employing CoO/NaCl or MnO/NaCl as dopant precursors respectively, while the other experimental conditions remain unchanged (Figures S7, S8a, and S10a, Supporting Information). In analogy to the research routes and characterization methods adopted above for Fe‐MoS_2_, XPS and EDX elemental mapping were conducted, identifying the existence of the heteroatoms (Figures S8f, S9, S10f, and S11, Supporting Information). More intriguingly, the electronic structure and phononic properties of pristine monolayer MoS_2_ can be similarly altered by doping with Co or Mn as proved by Raman, PL and XPS measurements (Figures S8b–e and S10b–e, Supporting Information). Therefore, from these results above, it can be inferred that this molten‐salt‐assisted doping strategy is an effective method for synthesizing doped monolayer MoS_2_ and altering its electronic structure and phononic properties, which provides opportunities for researching the doping‐related novel phenomenon of TMDs and further extending their applications in the future.

## 3 Conclusions

In summary, we develop a distinctive MACVD strategy to promote the volatilization of the dopant precursors for making it match with the growth process of monolayer MoS_2_, thus realizing effective doping with transition metal Fe. Besides, the presence of the Fe dopants is proved by XPS and HADDF‐STEM measurements. Compared with the pristine MoS_2_ nanoflakes, the electronic structures of Fe‐MoS_2_ have been altered by the Fe dopants evidenced by PL, STM, and XPS characterizations. It should be noted that this n‐type doping will weaken the bond strength of Mo‐S, resulting in the redshift of both A'_1_ and E' peaks. Moreover, the temperature‐dependent Raman spectrum is employed for the first time to explore the effect of the Fe dopants on the first‐order temperature coefficient of MoS_2_, and the linear temperature coefficients *χ*_T_ of Fe‐MoS_2_ nanoflakes become larger than that of the pristine MoS_2_. Based on theoretical calculation, this phenomenon can be attributed to the increased anharmonicity in the electron‐doped MoS_2_, suggesting that the phononic properties of monolayer MoS_2_ can also be modified by Fe‐doping. In addition, Co or Mn‐doped monolayer MoS_2_ nanoflakes can also be synthesized through the same way. This work presents an intriguing and powerful doping strategy for TMDs and paves the way for extending their applications in the area of spintronics, valleytronic, and photoelectric devices.

## 4 Experimental Section

##### Sample Preparation

The transition metal‐doped monolayer MoS_2_ nanoflakes were achieved by MACVD inside a multitemperature‐zone tubular furnace (Lindberg/Blue M) equipped with a 1 in. diameter quartz tube. By taking Fe doped MoS_2_ as an example, MoO_3_ (30 mg) was placed in the first zone of the furnace, and then the mixture of Fe_3_O_4_ and NaCl (mixed at the mass ratio of 1:2, 20 mg) was put just upstream of the sapphire (0001) substrates in the second zone of the furnace. In the growth process, the chamber was kept in low pressure (30 Pa), and when the temperature of the first zone reached 400 °C, S vapors were introduced into the furnace with heating belts at 110 °C with argon. Typical growth conditions were optimized at a carrier gas flow rate of ≈50 sccm, the temperature of the first zone of furnace ≈530 °C, while the temperature of the second zone of furnace ≈870 °C. The furnace was heated from room temperature to target temperature within 30 min, and the growth time was set at 15 min. Once the heating process ended, the furnace was allowed to cool down naturally. As for the synthesis of Co or Mn‐doped monolayer MoS_2_, Fe_3_O_4_ powder was replaced by CoO or MnO, other experimental conditions remained unchanged.

##### Transfer of Samples

The as‐grown Fe‐MoS_2_ and pure MoS_2_ samples were transferred to Cu grids or other substrates with the aid of polymethyl methacrylate (PMMA). Specifically, the sample was first spin‐coated with PMMA at a speed of 2000 rpm for 1 min followed by baked at 120 °C for 5 min, and then the edge of PMMA film was scraped with a tweezer to provide a channel for the water penetration. Afterward, the hot water was used to detach MoS_2_ adlayer from sapphire, a fresh Cu grid or other substrates were then used to “fish out” the PMMA‐capped sample, followed by rinsing with acetone to remove the PMMA.

##### Characterizations of Samples

The monolayer Fe‐MoS_2_ and pure MoS_2_ nanoflakes were systematically characterized using optical microscopy (Nexcope NM910), AFM (Bruker, Dimension Icon), Raman and PL spectroscopy (Horiba, XploRA PLUS, excitation light ≈532 nm, the spectral resolutions ≈1.8 cm^−1^ with an 1800 grooves/mm grating, the positions of the characteristic Raman peaks were the statistical average values of three times measurements at different spots with Gaussian fitting), EDX spectrum and elemental mapping (FEI Tecnai F20), High‐resolution HADDF‐STEM (Titan Cubed Themis G2). XPS measurement was conducted by Kratos Analytical AXIS‐Ultra with monochromatic Al K*α* as X‐ray excitation source, besides the samples grown on the substrates can be tested directly and do not need to be treated particularly. STM characterization was performed by utilizing an Omicron ultrahigh vacuum STM under a base pressure better than 10^−9^ mbar at room temperature after transferring the 2D nanoflakes onto HOPG substrate with high conductivity.

##### Theoretical Calculations

Crystal orbital overlapping population (COOP)^[^ ^45^, ^46^ ^]^ was used to evaluate all pair‐wise interactions between the *i*th and *j*th atomic orbitals at *R* and *R*_0_ sites by the product of the overlap matrix *S*_*iR*,*jR*′_ and electronic density of states

(1) $$\mathrm{COOP}\;\left(\varepsilon \right)=\sum_{nk}f_{nk}c_{nk}^{iR*}S_{iR,jR^{\prime }}c_{nk}^{jR^{\prime }}\delta \left(\varepsilon_{nk}-\varepsilon \right)\;$$

in which *n* is the index of the electronic band, *k* is the wave vector, *f*_nk_ is the distribution function satisfying the Fermi–Dirac statistics, and *ε*_nk_ is the electronic band. $\Psi_{\mathrm{nk}}=\sum_{iR}c_{nk}^{iR}\phi_{iR}\;$ is the system wave function summing over the *i*th atomic orbital *φ*_*iR*_ at the *R* site. The overlap matrix *S*_*iR*,*jR*′_ = 〈*φ*_*iR*_|*φ*_*jR*′_〉  arises from the *i*th and *j*th atomic orbitals at *R* and *R’*. Positive, negative, and zero values of COOP represent respectively the bonding, anti‐bonding and non‐bonding states.

Inter‐atomic potential was obtained by using density functional theory (DFT) as implemented in the Quantum Espresso package.^[^ ^47^ ^]^ The Perdew–Zunger functional^[^ ^48^ ^]^ within the local‐density approximation (LDA) was used to describe electronic exchange‐correlation interaction and norm‐conserving pseudopotentials to describe the interaction between valence electrons and nucleus. A Monkhorst‐Pack k grid^[^ ^49^ ^]^ of 8 × 8 × 1 was used to sample the Brillouin zone (BZ) of MoS_2_ for total energy calculations. The electronic kinetic energy cutoff of 65 Ry was chosen for the plane‐wave basis and total energy difference no more than 10^−10 ^eV between the two self‐consistency iterations was set as the self‐consistency criterion. The non‐resonant Raman spectra of MoS_2_ was calculated based on the Placzek approximation.^[^ ^50^ ^]^

## Conflict of Interest

The authors declare no conflict of interest.

## Supporting information

Supporting Information
