# Supplementary material for: Molten‐Salt‐Assisted Chemical Vapor Deposition Process for Substitutional Doping of Monolayer MoS2 and Effectively Altering the Electronic Structure and Phononic Properties
Source: Adv Sci (Weinh). 2020 Jul 1;7(16):2001080. doi: 10.1002/advs.202001080 (PMC7435234; doi:10.1002/advs.202001080)
Supplement: Supplementary file 1 — Supporting Information [file ADVS-7-2001080-s001.pdf]

WILEY-VCH

Copyright WILEY-VCH Verlag GmbH & Co. KGaA, 69469 Weinheim, Germany, 2018.

Supporting Information

Molten-Salt-Assisted Chemical Vapor Deposition Process for Substitutional Doping of Monolayer MoS_2 and Effectively Altering the Electronic Structure and Phononic Properties

*Wei Li, Jianqi Huang, Bo Han, Chunyu Xie, Xiaoxiao Huang, Kesong Tian, Yi Zeng, Zijing Zhao, Peng Gao, Yanfeng Zhang, Teng Yang, Zhidong Zhang, Shengnan Sun, and Yanglong Hou**

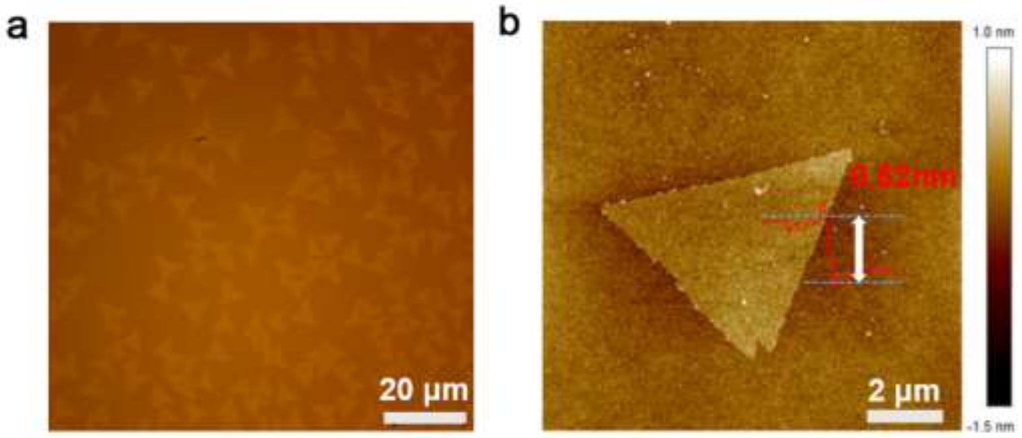

Figure S1. a) Optical image of the obtained pure MoS_2 nanoflakes. b) AFM image and the height profile of pure MoS_2 nanoflake.

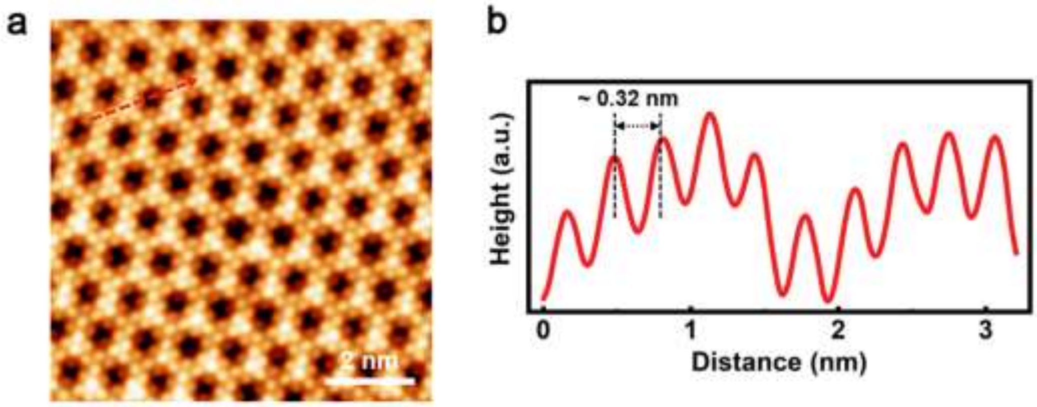

Figure S2. a) Scanning tunneling micrograph of pure monolayer MoS_2 on HOPG. b) Corresponding line profiles along the arrows in (a).

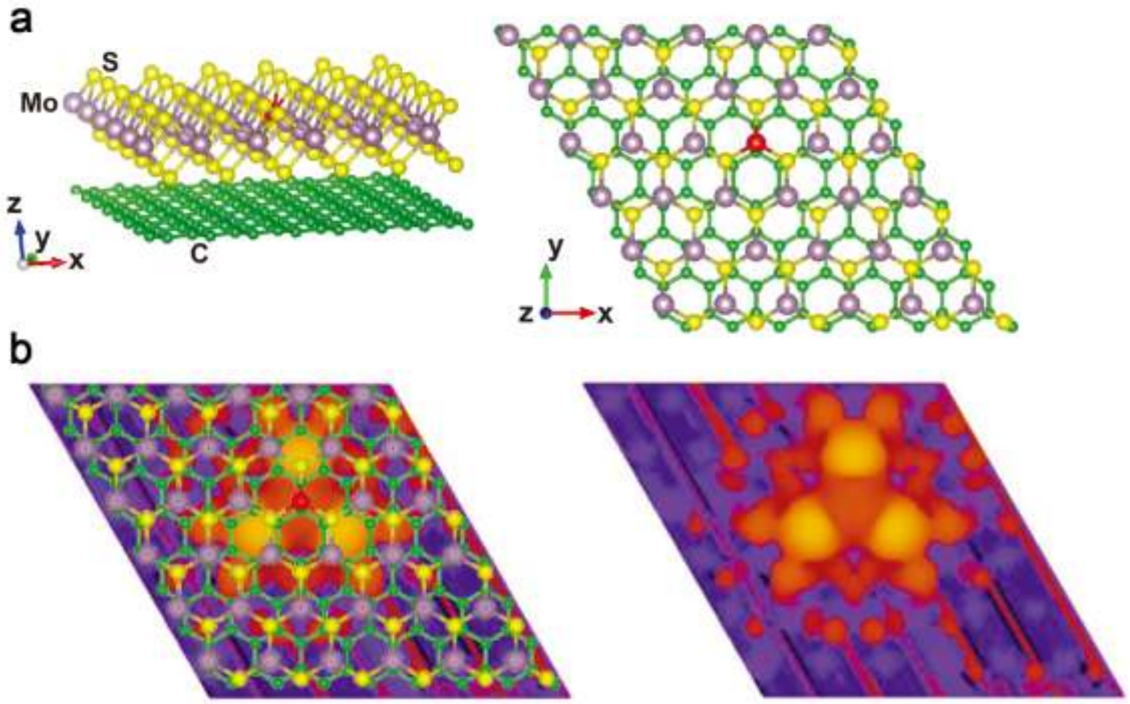

Figure S3. a) Structure of Fe-MoS_2 on graphene. The tilted and top views are on the left and right side, respectively. b) Calculated STM image. Left image is with atomic structure superposed on top. We used a 6 × 6 MoS_2 supercell and 8 × 8 graphene supercell, with one Mo atom substituted by Fe (in red). STM image was calculated on the local density of states calculation (LDOS) using the constant current mode in the Tersoff-Hamann approximation. The STM charge density was calculated within the energy range from E_F to E_F + 0.55 eV (corresponding to -0.55 V bias voltage in the experiment) and the iso-surface of electron density was set to be 10^{-4} e/Bohr^3.

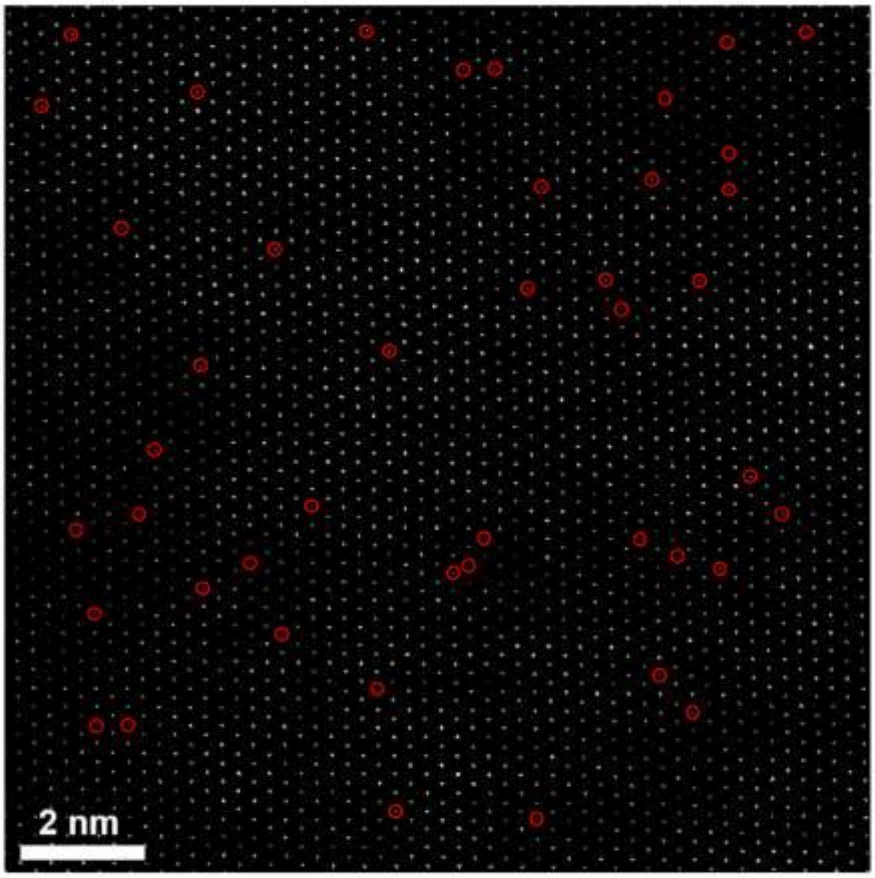

Figure S4. High-resolution HADDF-STEM image of Fe-MoS_2 and Fe atoms are partly remarked by red circles based on their lower intensity in HAADF compared with Mo atoms.

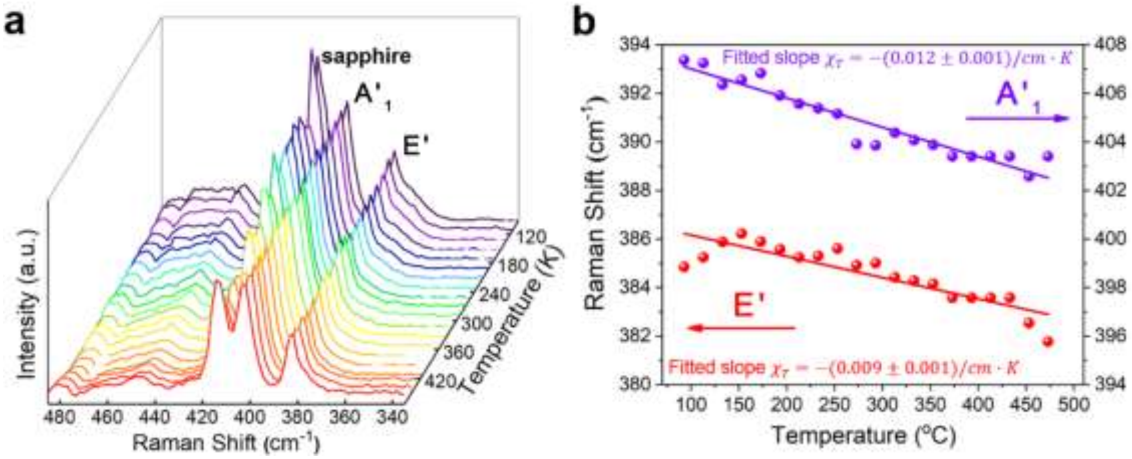

Figure S5. a) Raman spectra of monolayer MoS_2 collected at various temperatures between 93 K to 473 K. b) Raman peak frequencies of both A'$_1$ (violet circles) and E' (red circles) modes as a function of temperature.

Table S1. The corresponding specific Raman shift of pristine MoS_2, Mn-MoS_2, Fe-MoS_2, and Co-MoS_2 concluded from Figure 2, S6 and S8

Vibrational Mode	Pristine MoS_2	Mn-MoS_2	Fe-MoS_2	Co-MoS_2
E' [cm^{-1}]	385.4	384.4	383.8	383.6
A'_1 [cm^{-1}]	405.2	404.3	404.1	403.8

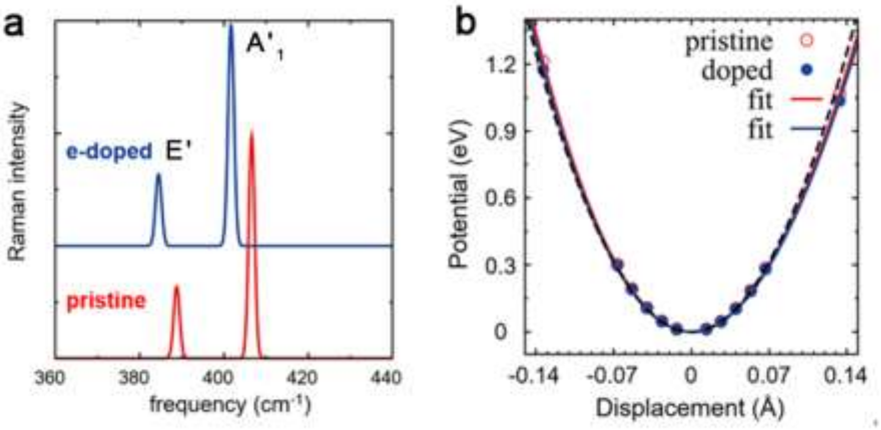

Figure S6. a) The calculated Raman spectra of both pristine (red) and electron-doped (blue) MoS_2. 0.05 electrons per formula unit are used to simulate the electron-doped system. b) Vibrational potential vs. atomic displacement curves of the E' mode. Dots are the calculation data, and solid lines are the fitting using the Morse potential. The dashed black line is for a perfect harmonic potential.

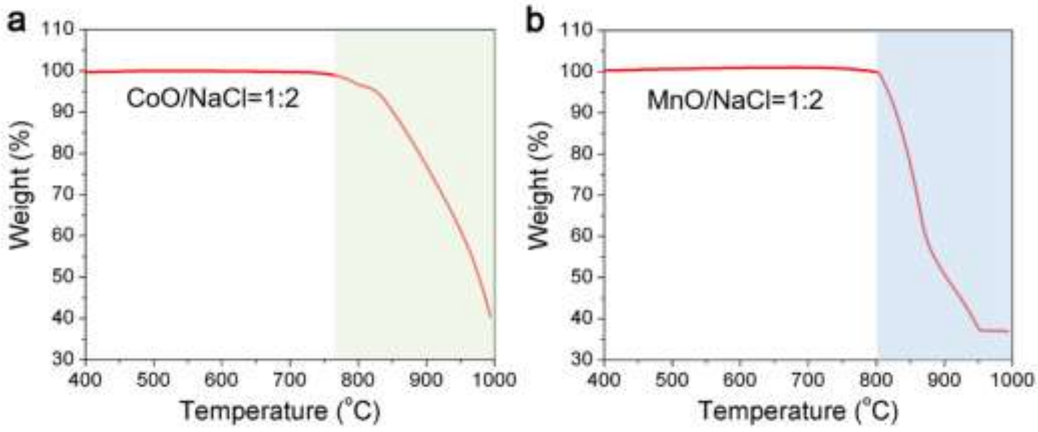

Figure S7. The thermogravimetric curve of the CoO/NaCl (a) and MnO/NaCl (b) at the mass ratio of 1:2.

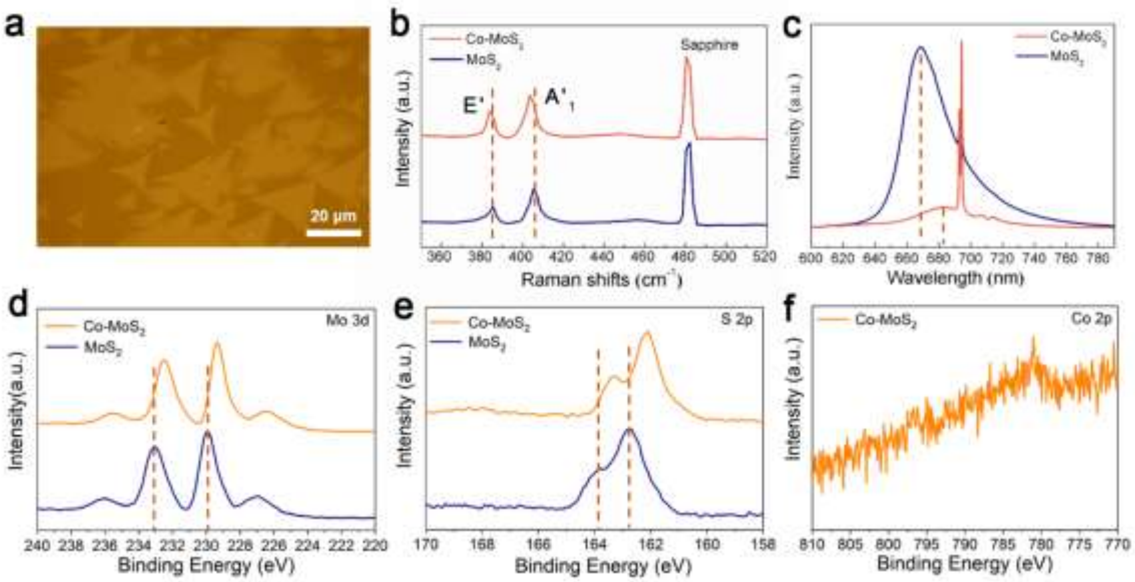

Figure S8. a) Optical image of the obtained Co-MoS_2 nanoflakes. b, c) Raman and PL spectra of Co-MoS_2 and pure MoS_2, respectively. d, e) XPS scans of Mo 3d (d), S 2p (e), core-level binding energies of Co-MoS_2 and pure MoS_2, calibrated by the adventitious carbon C 1s peak. f) XPS scans of Co 2p core-level binding energy of Co-MoS_2.

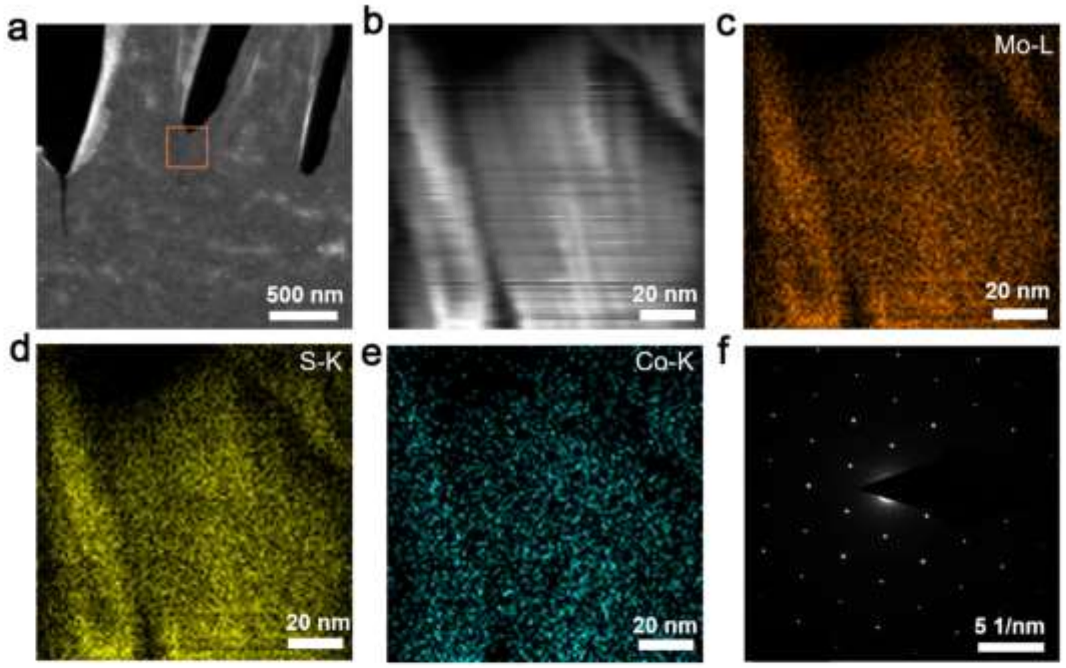

Figure S9. a, b) HAADF-STEM image of Co-MoS_2 nanoflakes transferred onto Cu grids. c-e) EDX elemental mapping images of the sample shown in (b). f) Corresponding selected area electron diffraction pattern of Co-MoS_2 nanoflakes.

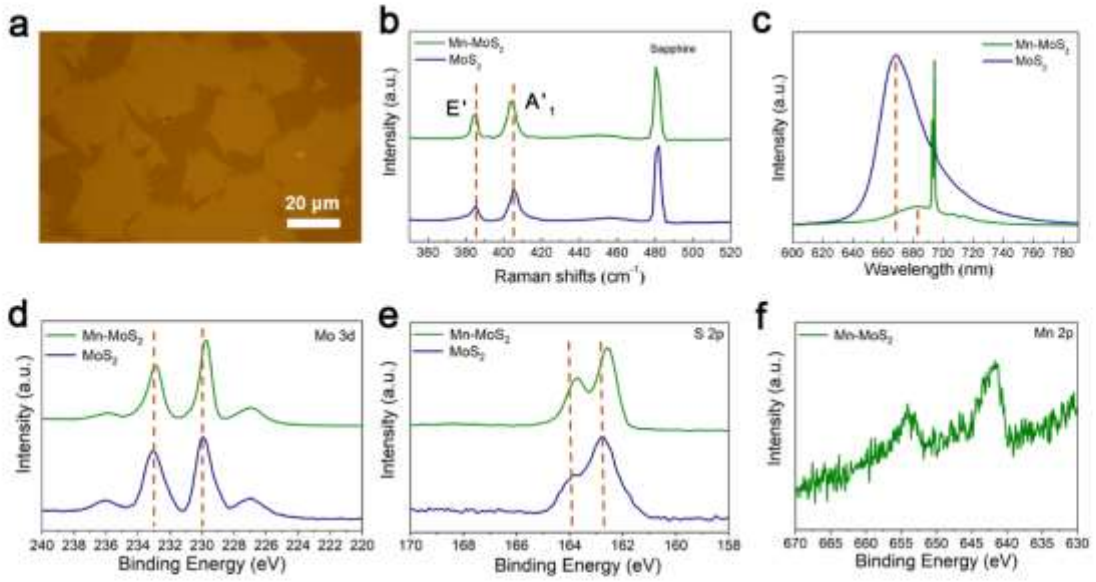

Figure S10. a) Optical image of the obtained Mn-MoS_2 nanoflakes. b, c) Raman and PL spectra of Mn-MoS_2 and pure MoS_2, respectively. d, e) XPS scans of Mo 3d (d), S 2p (e), core-level binding energies of Mn-MoS_2 and pure MoS_2, calibrated by the adventitious carbon C 1s peak. f) XPS scans of Mn 2p core-level binding energy of Mn-MoS_2.

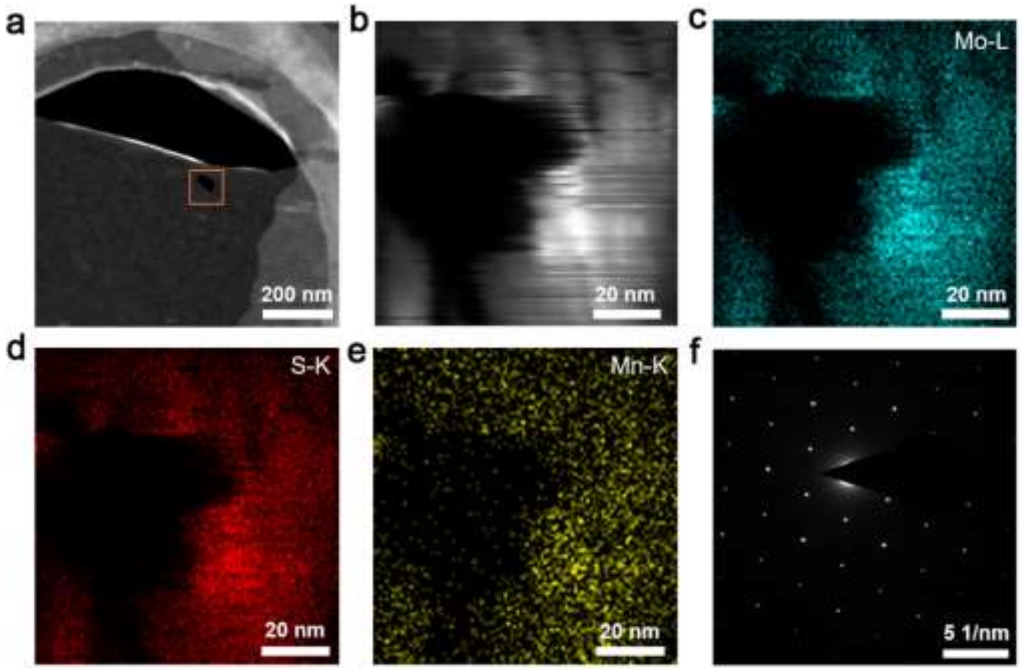

Figure S11. a, b) HAADF-STEM images of Mn-MoS_2 nanoflakes transferred onto Cu grids. c-e) EDX elemental mapping images of the sample shown in (b). f) Corresponding selected area electron diffraction pattern of Mn-MoS_2 nanoflakes.
